# Identification of a Novel Mesenchymal Stem Cell–Related Signature for Predicting the Prognosis and Therapeutic Responses of Bladder Cancer

**DOI:** 10.1155/sci/6064671

**Published:** 2024-11-15

**Authors:** Enguang Yang, Luhua Ji, Xinyu Zhang, Suoshi Jing, Pan Li, Hanzhang Wang, Luyang Zhang, Yuanfeng Zhang, Li Yang, Junqiang Tian, Zhiping Wang

**Affiliations:** ^1^Institute of Urology, Key Laboratory of Gansu Province for Urological Diseases, Gansu Urological Clinical Center, Lanzhou University Second Hospital, Lanzhou 730030, China; ^2^Department of Pathology and Laboratory Medicine, Legorreta Cancer Center at Brown University, The Warren Alpert Medical School of Brown University, Brown University Health, Providence 02912, Rhode Island, USA

**Keywords:** mesenchymal stem cell, tumor microenvironment, urinary bladder, urothelial carcinoma

## Abstract

**Background:** Mesenchymal stem cells (MSCs) have been identified to have a unique migratory pattern toward tumor sites across diverse cancer types, playing a crucial role in cancer progression, treatment resistance, and immunosuppression. This study aims to formulate a prognostic model focused on MSC-associated markers to efficiently predict the clinical outcomes and responses to therapy in individuals with bladder cancer (BC).

**Methods:** Clinical and transcriptome profiling data were extracted from The Cancer Genome Atlas Urothelial Bladder Carcinoma (TCGA-BLCA) and GSE31684 databases. Systematic quantification of MSC prevalences and stromal indices was undertaken, culminating in the discernment of genes correlated with stromal MSCs following a thorough application of weighted gene coexpression network analysis techniques. Subsequently, an exhaustive risk signature pertinent to MSC was formulated by amalgamating methods from univariate and Least Absolute Shrinkage and Selection Operator (LASSO) Cox regression models. Drugs targeting genes associated with MSCs were screened using molecular docking.

**Results:** The prognostic model for MSC incorporated five critical genes: ZNF165, matrix remodeling-associated 7 (MXRA7), CEMIP, ADP-ribosylation factor-like 4C (ARL4C), and cerebral endothelial cell adhesion molecule (CERCAM). In the case of BC patients, stratification was performed into discrete risk categories, utilizing the median MSC risk score as a criterion. It was striking that those classified within the high-MSC-risk bracket demonstrated correlations with unfavorable prognostic implications. Enhanced responsiveness to immunotherapy in low-MSC-risk patients was delineated compared to their high-MSC-risk counterparts. A heightened receptivity was noted toward particular chemotherapy drugs, encompassing gemcitabine, vincristine, paclitaxel, gefitinib, and sorafenib, within this high-risk group. Conversely, a superior reaction to cisplatin was distinctly evident among those marked by low MSC scores. The results of molecular docking demonstrated that kaempferol exhibited favorable docking with ZNF165, quercetin exhibited favorable docking with MXRA7, mairin exhibited favorable docking with CEMIP, and limonin diosphenol exhibited favorable docking with ARL4C.

**Conclusions:** The five-gene MSC prognostic model demonstrates substantial efficacy in prognosticating clinical outcomes and gauging responsiveness to chemotherapy and immunotherapy regimens. The genes ZNF165, MXRA7, CEMIP, ARL4C, and CERCAM are underscored as promising candidates warranting further exploration for anti-MSC therapeutic strategies, thereby offering novel insights for personalized treatment approaches in BC.

## 1. Introduction

Globally, bladder cancer (BC) stands as the tenth most prevalent form of cancer, impacting individuals across all gender identities. In the year 2020 alone, it was calculated that BC was the diagnosis given in ~573,000 new cancer cases while also being responsible for roughly 12,000 deaths [1]. The tumor microenvironment (TME) in BC is characterized by its dynamic nature, marked by significant alterations and complex intercellular dialogues during phases of tumor development, metastatic expansion, and responses to treatments. Consequently, BC is characterized as a complex biological network, inclusive of an array of nonmalignant cells and their intricate interactions embedded within the tumor matrix [2]. Despite this complexity, predominant research efforts have been focused on exploring the genetic and epigenetic modifications occurring within the urothelial cell clusters. There arises an unequivocal need to delve into the influence exerted by the stromal segment of the TME on the evolution and progression of BC. This amplified focus on the cellular and molecular activities within the urothelial cells has inadvertently led to a gap in understanding the comprehensive landscape of BC. A nuanced exploration into the roles and impacts of the stromal compartment, especially in the context of its interactions with cancerous cells and contributions to the TME's dynamism, could unveil novel insights into BC pathophysiology and potential therapeutic interventions.

Mesenchymal stem cells (MSCs) are noted for their distinct ability to home to various tumor types, significantly influencing tumor progression [3, 4]. In colon cancer, MSCs enhance stemness and epithelial–mesenchymal transition through interleukin-8 (IL-8)/CXC chemokine receptor 2 (CXCR2)/mitogen-activated protein kinase (MAPK) and fibroblast growth factor 10 (FGF10)–protein kinase A (PKA)–protein kinase B (Akt)–β-catenin signaling pathways [5]. A substantial body of evidence, supported by both in vitro and in vivo studies, confirms the role of MSCs in engendering resistance to chemotherapy and radiotherapy [6, 7]. Mitochondrial transfer from adipose MSCs to cancer cells occurs via tunneling nanotubes and contributes to multidrug resistance [8, 9]. Carcinoma-associated MSCs (CA-MSCs) further enhance ovarian cancer metastasis by donating mitochondria to “mito-poor” tumor cells, thereby restoring their proliferative capacity and chemotherapy resistance [10]. Additionally, the activation of CXCR1/Akt signaling induced by MSC-derived IL-8 promotes anoikis resistance and pulmonary metastasis in osteosarcoma cells [11]. Furthermore, MSC-derived RAB22A-induced extracellular vesicles (EVs) containing activated stimulator of interferon genes (STING) can effectively induce antitumor immunity by promoting interferon beta (IFNβ) expression in monocytes and enhancing immune responses [12]. The presence of MSCs has been confirmed not only in human bladder tissue but also in urine, highlighting their pervasive nature [13–15]. Conditioned media from MSCs are rich in chemokine ligand 2 (CCL2)/monocyte chemoattractant protein-1 (MCP-1), matrix metalloproteinases (MMPs), and ILs, which promote tumor aggressiveness and chemoresistance in BC cells [16]. In light of these findings, delving into the implications of stromal MSC-associated factors within the sphere of BC is deemed essential.

Targeting the TME through innovative treatments is recognized as a promising strategy in combating cancer. This approach is advantageous because it acts on genetically stable cells, mitigating the risk of developing drug resistance [17]. Prior research has illuminated the potential of inhibiting stromal components within the TME to arrest tumor advancement [18–20]. A holistic and system-level approach, encompassing diverse therapeutic targets within the TME, is essential for optimizing antitumor results [17]. However, the field lacks extensive studies on identifying and developing therapeutic targets specifically for MSCs.

In the present study, a unique five-gene MSC signature was developed. The constructed model exhibited predictive capabilities for both prognosis and therapeutic responses. Meanwhile, the effective drugs that targeted MSC-related genes were screened by molecular docking. Evidence indicates that this MSC-based model could pave the way for new therapeutic interventions targeting MSCs within the BC landscape.

## 2. Materials and Methods

### 2.1. Data Retrieval and Preparation

Clinical and RNA sequencing (RNAseq) data, harmonized to fragments per kilobase million (FPKM), were retrieved from the Bladder Urothelial Carcinoma (BLCA) dataset within the Cancer Genome Atlas (TCGA) database (https://portal.gdc.cancer.gov/). Criteria for valid data inclusion involved the exclusion of entries with a follow-up time documented as 0 days and the removal of repeated datasets. Annotation was facilitated by gene transfer format (GTF) files sourced from Ensemble (http://asia.ensembl.org), essential for distinguishing between messenger RNAs (mRNAs) and long non-coding RNAs (lncRNAs) in the proceeding stages of analysis. A separate set of normalized expression data and clinical records, associated with 93 BC samples, was acquired from the Gene Expression Omnibus (GEO) database, specifically referencing GSE31684 [21]. In instances of multiple probes correlating with a single gene, prioritization was given to the probe exhibiting the highest value.

### 2.2. Estimation of MSC Infiltration and Calculation of Stromal Score

MSC abundances were ascertained utilizing the xCell algorithm, grounded in gene signature enrichment [22]. This computational tool aids in delivering a nuanced understanding of the intricate cellular composition within the tumor environment. In parallel, the analysis employed the ESTIMATE algorithm, facilitated through the estimate R package (version 4.2.0), to discern the stromal score [23]. This metric serves as a barometer for evaluating stromal infiltration levels within individual tumor specimens, offering insights into the complex interplay between diverse cell types and the TME.

### 2.3. Construction of MSC and Stromal Coexpression Networks

The study employed the WGCNA R package (version 1.68) [24] for constructing coexpression networks, aiming to identify key genes associated with MSC infiltrations and stromal scores. By analyzing data extracted from TCGA-BLCA and GSE31684 cohorts, the focus was directed toward a specific subset of 5000 genes, particularly those distinguished by a substantial median absolute deviation (MAD). This intricate process involved the creation of a similarity matrix, sij, derived from Pearson's correlation calculations between gene pairs. A refined step in this methodology was the augmentation of the matrix using a soft-thresholding power β (sij β), a strategic move to comply with the scale-free topology criterion. Following this, the altered matrix, referred to as an adjacency matrix, underwent classification through the utilization of the topological overlap measure (TOM) and the complementary dissimilarity (1-TOM) concerning genes. The division of modules from the subsequent dendrogram was achieved via the dynamic tree-cut method, ensuring each module comprised at least 30 genes. Calculations of module eigengenes (MEs) were conducted, extracted from the principal component that encapsulates the gene expression profiles within each module. This phase was succeeded by an exhaustive correlation scrutiny between MEs, MSC infiltrations as assessed by xCELL, and the respective stromal scores. Modules were prioritized based on the strength of their correlation for additional analysis. In the module of interest, intense examination was conducted on both gene significance (GS) for traits and module membership (MM), thereby highlighting the connection between MEs and specific gene expressions. The identification of hub genes was stringent, requiring a GS beyond 0.4 and an MM surpassing 0.8. Validating the credibility of these hub genes necessitated a parallel investigation within the TCGA-BLCA and GSE31684 cohorts, solidifying the final selection.

### 2.4. Analysis of Gene Ontology (GO) and the Kyoto Encyclopedia of Genes and Genomes (KEGG)

The definitive group of hub genes was analyzed for GO and KEGG pathway enrichment using the clusterProfiler R package (version 3.14.3) [25]. This comprehensive analysis was instrumental in deciphering the vast array of biological roles, shedding light on the intricacies of biological processes (BPs), molecular functions (MFs), and cellular components (CCs). Furthermore, it helped identify the specific enriched pathways tied to these genes. A stringent criterion was applied for the enrichment analysis, accepting only results with a *p*-value of less than 0.05, emphasizing the most impactful pathways and functions. This method provided an in-depth understanding of the significant roles these hub genes assume within various biological contexts.

### 2.5. Construction and Validation of Prognostic Model

The selection of the TCGA-BLCA cohort was intentional for creating the MSC risk model, while the GSE31684 cohort, consisting of 93 cases, served as the verification group. The initial step in this process involved the application of the univariate Cox regression model, instrumental in pinpointing stromal MSC hub genes with the potential as reliable prognostic markers for overall survival (OS). Criteria for selection stipulated a *p*-value below 0.05. The genes meeting this criterion underwent a Least Absolute Shrinkage and Selection Operator (LASSO) Cox regression analysis, facilitated by the glmnet R package. This iteration, repeated 1000 times, aimed to minimize the gene pool. The MSC risk model's establishment was anchored in deriving the MSC risk score, calculated by summing the products of LASSO coefficients (βi) and the expression levels (Expi) of individual genes. Patient categorization into elevated or reduced MSC-risk classifications hinged on median MSC risk scores. Kaplan–Meier curves and the log-rank test were instrumental in evaluating the variation in OS between these distinct classifications. The TCGA-BLCA cohort served a pivotal role in validating the efficacy and reliability of the constructed MSC risk model.

### 2.6. Collection of MSC Markers and Correlation Analysis

Markers specific to MSCs, as well as nonspecific ones, were identified from existing published resources [26]. In assessing the robustness and uniformity of specific markers in BC scenarios, a meticulous evaluation was performed using Spearman's correlation methods. This rigorous approach concentrated on deciphering the association between the risk scores of MSC and the scores derived from stromal analysis. In addition, this study delved into a detailed exploration, aiming to uncover the potential linkages among genes selected for the MSC paradigm and markers of MSC that pre-existing studies have acknowledged. By adopting such methodical techniques, this scrutiny not only underlined the significance of consistent markers within BC's realm but also fortified the foundational understanding of MSC role reflected through its risk and stromal scores. The intricate relationship between the selected gene constituents within the MSC framework and traditional MSC indicators was further elucidated, offering new insights and reinforcing the credibility of these markers in the context of BC research. This assessment was systematically carried out within both TCGA-BLCA and GSE31684 cohorts to ensure a thorough and inclusive analysis.

### 2.7. Prediction of Chemotherapy and Immunotherapy Response

Data procurement was executed from the Genomics of Drug Sensitivity in Cancer (GDSC) repository, available at https://www.cancerrxgene.org/ [27]. Determination of half-maximal inhibitory concentration (IC50) metrics, relevant to a cohort of drugs, namely, bleomycin, lapatinib, paclitaxel, camptothecin, cisplatin, docetaxel, methotrexate, and sunitinib, for each gastric cancer (GC) instance, utilized ridge regression methodology. Enhancement of this procedure involved tenfold cross-validation, courtesy of the pRRophetic R software [28, 29], underpinned by a thorough dissection of transcriptome datasets. Analytical refinement continued with the integration of the tumor immune dysfunction and exclusion (TIDE) web-based computation resource, located at http://tide.dfci.harvard.edu/, crucial for prognostically interpreting immunotherapy response variances through immune checkpoint blockade [30]. Discrepancies in response statistics between subsets categorized under high- versus low-MSC-risk underwent rigorous scrutiny via the chi-squared methodology. Subsequently, an exhaustive critique employing receiver operating characteristic (ROC) trajectories and the affiliated area under the metric (AUC) quantifications further substantiated the predictive precision and steadfastness of the MSC risk stratagem.

### 2.8. Enrichment Analyses

The exploration of gene set enrichment within TCGA-BLCA, particularly in groups with high- or low-MSC-risk, was conducted via gene set enrichment analysis (GSEA), leveraging the capabilities of enrichplot and clusterProfiler R packages. For this analysis, gene sets such as “c2.cp.kegg.v7.4.symbols” and “h.all.v7.4.symbols” were sourced from MSigDB [31]. Utilization of the single-sample GSEA (ssGSEA) methodology [32] facilitated the computation of enrichment scores for specific gene sets, including those involved in complement and coagulation cascades, extracellular matrix (ECM)–receptor interaction, and focal adhesion. A meticulous process was followed, involving Spearman's correlation analysis, to delve into the relationship between MSC risk scores and the scores derived from gene set enrichment. This approach illuminated the complex mechanisms and potential functional ramifications linked to varying degrees of MSC-related risk within BLCA.

### 2.9. Validation Using the Human Protein Atlas (HPA) Databases

For the exploration at the protein echelon, visual data corresponding to immunohistochemical (IHC) staining relative to specific markers in BC tissues were sourced from the HPA digital platform, accessible via https://www.proteinatlas.org/ [33]. This repository plays a critical role in the detailed visual examination of both the spatial distribution and abundance of targeted proteins present within the collected tissue samples. By systematically engaging with this strategy, there is an enhancement in the holistic understanding of the functional relevance and possible implications these proteins harbor within the BC framework.

### 2.10. Screening of Targeting Related Drugs and Molecular Docking

The Symptom Mapping (symMap) database was utilized in this study to forecast potential herbs that target genes related to MSCs. Herbs exhibiting a false discovery rate (FDR) below 0.05 were chosen. The composition of the identified drugs was examined using the Traditional Chinese Medicine Systems Pharmacology (TCMSP) online database, and components possessing an oral bioavailability (OB) of at least 30% and a drug likeness (DL) value of at least 0.18 were selected for further investigation. Prior to docking the two structures, ligand and receptor structures were obtained. Initially, the protein database (Uniprot; https://www.uniprot.org/) was accessed to retrieve essential protein backbone structures. The Pub Chemical Database (https://pubchem.ncbi.nlm.nih.gov/) was consulted to examine small molecule drug structures of compounds exhibiting notable OB values. Subsequently, the Research Collaboratory for Structural Bioinformatics Protein Data Bank (RCSB PDB) online tool (https://cadd.labshare.cn/cb-dock2/php/index.php) was employed for molecular docking. The most stable docking structures were chosen for further investigation.

## 3. Results

### 3.1. The Association Between Higher MSC Infiltrations and Stromal Scores and Poorer OS in BC Patients

**Figure 1:**
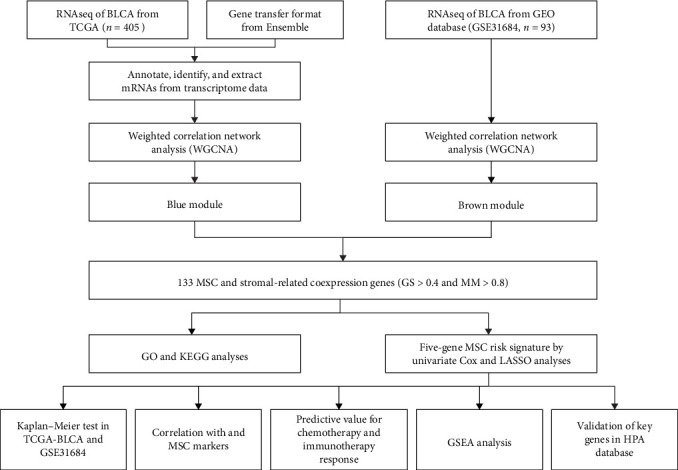
The flowchart of this research. GEO, Gene Expression Omnibus; GO, Gene Ontology; GS, gene significance; GSEA, gene set enrichment analysis; HPA, Human Protein Atlas; KEGG, Kyoto Encyclopedia of Genes and Genomes; LASSO, Least Absolute Shrinkage and Selection Operator; MM, module membership; mRNAs, messenger RNAs; MSC, mesenchymal stem cell; RNAseq, RNA sequencing; TCGA-BLCA, The Cancer Genome Atlas Urothelial Bladder Carcinoma.

**Figure 2:**
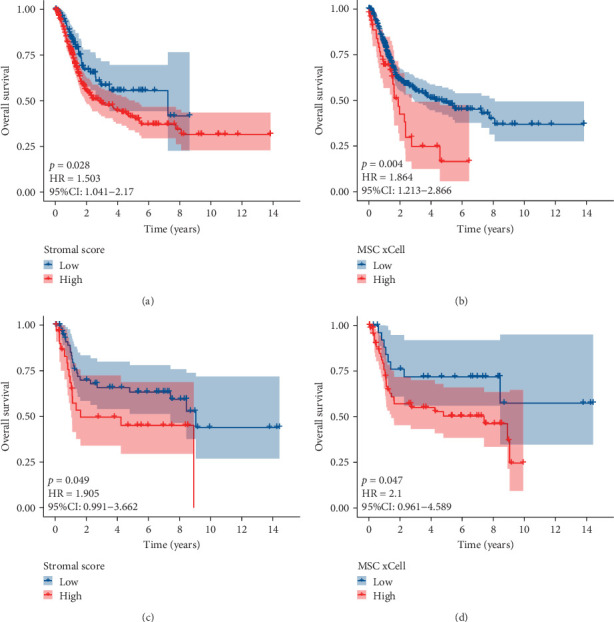
Kaplan–Meier curves showed that higher MSC infiltrations and stromal scores were significantly correlated with worse OS for bladder cancer patients in the TCGA-BLCA (A, B) and GSE31684 (C, D) cohorts. CI, confidence interval; HR, hazard ratio; MSC, mesenchymal stem cell; OS, overall survival; TCGA-BLCA, The Cancer Genome Atlas Urothelial Bladder Carcinoma.

The flowchart of the study is illustrated in Figure 1. MSC infiltration was assessed using xCellAnalysis, while the stromal score was determined using ESTIMATE. The predictive utility of these measures for OS was evaluated utilizing log-rank tests. Kaplan–Meier curves in TCGA-BLCA cohort (Figure 2A,B) and GSE31684 cohort (Figure 2C,D) showed a negative correlation between OS and higher MSC infiltration and stromal scores. Given these observations, an in-depth analysis of genes associated with MSC and stromal characteristics in BC was imperative.

### 3.2. The Coexpression Network Analysis of MSC and Stromal Scores

**Figure 3:**
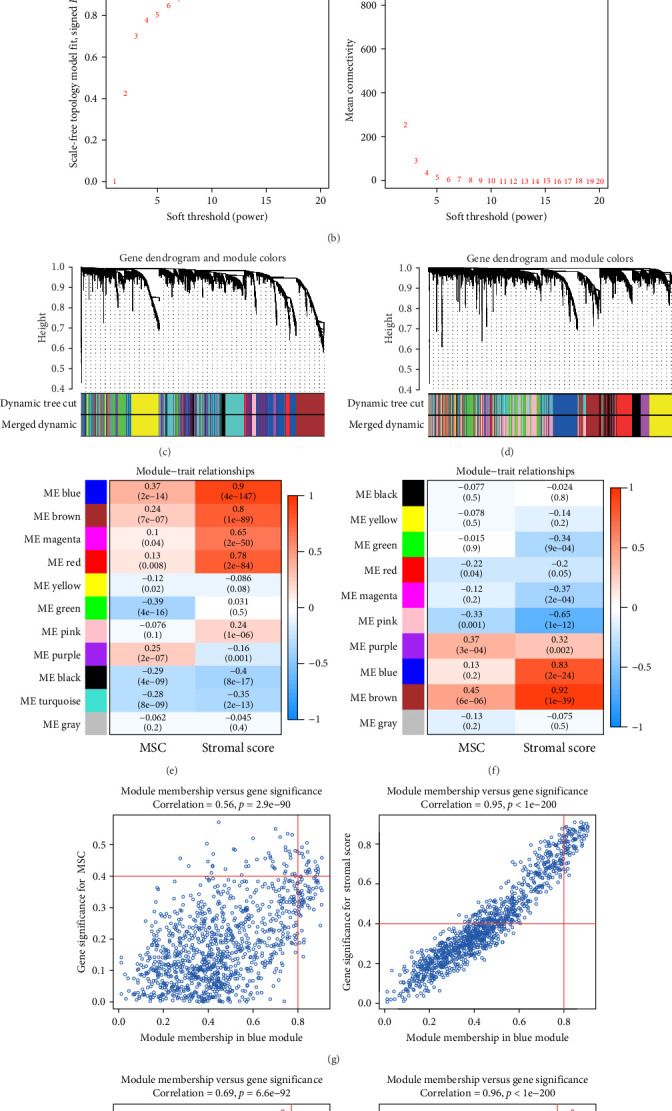
(A, B) Overview of the WGCNA methodology applied to TCGA-BLCA and GSE31684, with a soft threshold power (β) set at 4 and 8, respectively. (C, D) Dendrograms illustrating the clustering process, grouping genes with similar expression patterns into distinct coexpression modules for the TCGA-BLCA and GSE31684 datasets. (E, F) Analysis of module–trait relationships, highlighting the connections between individual gene module eigengenes and phenotypic traits specific to TCGA-BLCA and GSE31684. (G, H) Scatter plots visualizing the correlation between module membership (MM) and gene significance (GS) for the blue module in TCGA-BLCA and the brown module in GSE31684. The horizontal axis represents gene-to-module associations, while the vertical axis illustrates gene-to-phenotype correlations. ME, module eigengene; MSC, mesenchymal stem cell; TCGA-BLCA, The Cancer Genome Atlas Urothelial Bladder Carcinoma.

WGCNA analysis was conducted to dissect gene expression frameworks in the TCGA-BLCA and GSE31684 datasets. Specific soft threshold powers (four for TCGA-BLCA and eight for GSE31684) were applied to construct scale-free topology networks (Figures 3A,B). Hierarchical clustering trees revealed 11 unique coexpression patterns for TCGA-BLCA (Figure 3C), wherein the blue module exhibited a noteworthy positive association with MSC proportion (correlation = 0.37, *p* = 2e−14) and the stromal score (correlation = 0.90, *p* = 4e−147) (Figure 3E). Concurrently, the analysis of GSE31684 yielded 10 coexpression patterns (Figure 3D), with the brown module drawing attention for its robust positive association with MSC proportion (correlation = 0.45, *p* = 6e−6) and stromal score (correlation = 0.92, *p* = 1e−39) (Figure 3F). Encapsulated within these modules were 1081 genes for the blue and 560 for the brown, necessitating an in-depth analysis. This investigation of scatter plots pertaining to the blue module underscored stark correlations between MM and GS for MSC (correlation = 0.56, *p* = 2.9e−90) and stromal scores (correlation = 0.95, *p* < 1e−200) (Figure 3G). A correspondingly potent association was discerned in the brown module concerning MSC (correlation = 0.69, *p* = 6.6e−92) and stromal scores (correlation = 0.96, *p* < 1e-200) (Figure 3H). The strategic application of MM > 0.8 and GS > 0.4 as threshold parameters led to the recognition of 376 and 380 pivotal genes within the blue and brown modules of TCGA-BLCA and GSE31684, respectively. These genes are significantly intertwined with both MSC and stromal scores.

### 3.3. Enrichment Analyses Conducted on Hub Genes

**Figure 4:**
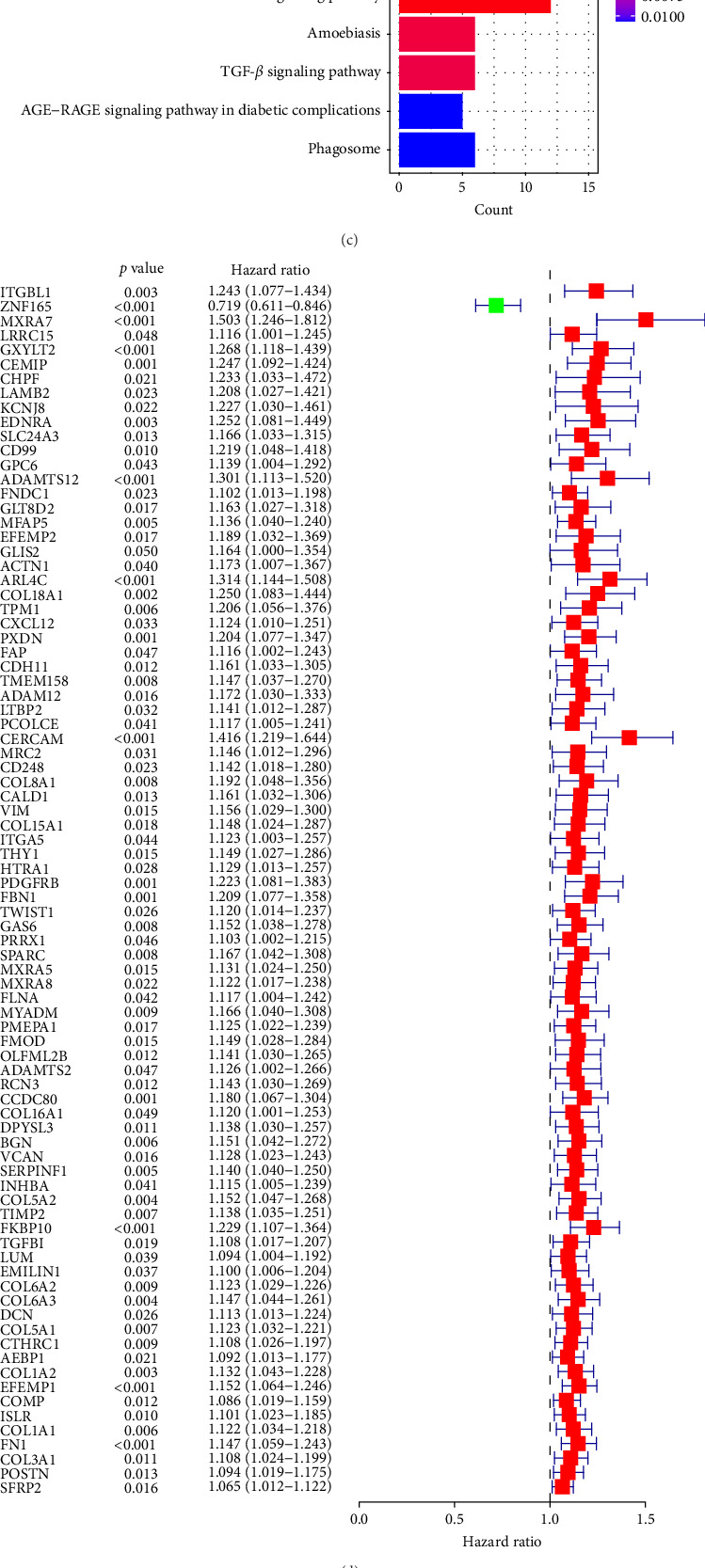
(A) Venn diagram illustrating the overlap of hub genes identified in the TCGA-BLCA blue module and the GSE31684 brown module. (B, C) Graphical representations of Gene Ontology (GO) analysis results, showcasing major themes in biological process (BP), cellular component (CC), and molecular function (MF) classifications (B), along with an exploration of KEGG pathways (C) focused on the 133 integral genes. (D) Univariate Cox analysis results, highlighting genes associated with overall survival (OS) in the TCGA-BLCA dataset. (E) Coefficient trajectories from LASSO Cox regression analysis, with the optimal adjustment parameter (λ) derived from partial likelihood deviance and supported by tenfold cross-validation. (F, G) Kaplan–Meier survival plots demonstrating a significant difference in OS between high-MSC-risk and low-MSC-risk groups, where the high-MSC-risk faction exhibits a markedly reduced OS compared to their low-risk counterparts, observed in both the TCGA-BLCA (HR = 2.435; 95% CI: 1.739–3.411; log-rank *p*  < 0.001) (F) and GSE32894 cohorts (HR = 2.233; 95% CI: 1.15–4.335; log-rank *p*=0.015) (G). AGE, advanced glycation end product; Akt, protein kinase B; CI, confidence interval; ECM, extracellular matrix; HR, hazard ratio; KEGG, Kyoto Encyclopedia of Genes and Genomes; LASSO, Least Absolute Shrinkage and Selection Operator; MSC, mesenchymal stem cell; PI3K, phosphoinositide 3-kinase; RAGE, receptor for advanced glycation end product; TCGA-BLCA, The Cancer Genome Atlas Urothelial Bladder Carcinoma; TGF, transforming growth factor; TGF-β, transforming growth factor beta.

A Venn diagram reveals the identification of 133 shared genes at the intersection of two distinct hub gene sets (Figure 4A). GO and KEGG analyses were performed on the 133 identified genes, revealing significant enrichment in BPs related to ECM organization, external encapsulating structure organization, and extracellular structure organization. Notably, collagen-containing ECM and ECM structural constituents were the major enriched terms in CCs and MFs, respectively (Figure 4B). The KEGG pathway analysis revealed significant enrichment in protein digestion and absorption, ECM–receptor interaction, and focal adhesion (Figure 4C).

### 3.4. Development of the Prognostic Risk Model Based on Stromal MSCs

TCGA-BLCA samples served as the training cohort, while samples from GSE31684 were utilized as the validation group. A univariate Cox regression analysis was performed on the 133 shared hub genes, identifying 84 genes significantly associated with OS with *p*-values less than 0.05. These genes were then subjected to LASSO Cox regression analysis (Figures 4D,E). From this analysis, five genes were identified for constructing the MSC risk model: MSC risk score = ZNF165 × (−0.1255) + MXRA7 × (0.1018) + CEMIP × (0.0329) + ARL4C × (0.0014) + CERCAM × (0.1569). BC patients from each cohort were stratified into high- and low-MSC-risk groups based on the median risk score. The Kaplan–Meier survival curves revealed that the high-MSC-risk group had significantly poorer OS compared to the low-MSC-risk group in both the TCGA-BLCA cohort (HR = 2.435; 95% CI: 1.739–3.411; log-rank *p*  < 0.001) (Figure 4F) and the GSE31684 cohort (HR = 2.233; 95% CI: 1.15–4.335; log-rank *p*=0.015) (Figure 4G). The findings of this study suggest that the expression of MSC and stromal-related signature genes plays a significant role as prognostic markers in BC.

### 3.5. Strong Correlation Was Observed Between MSC Signature Genes and MSC Markers

**Figure 5:**
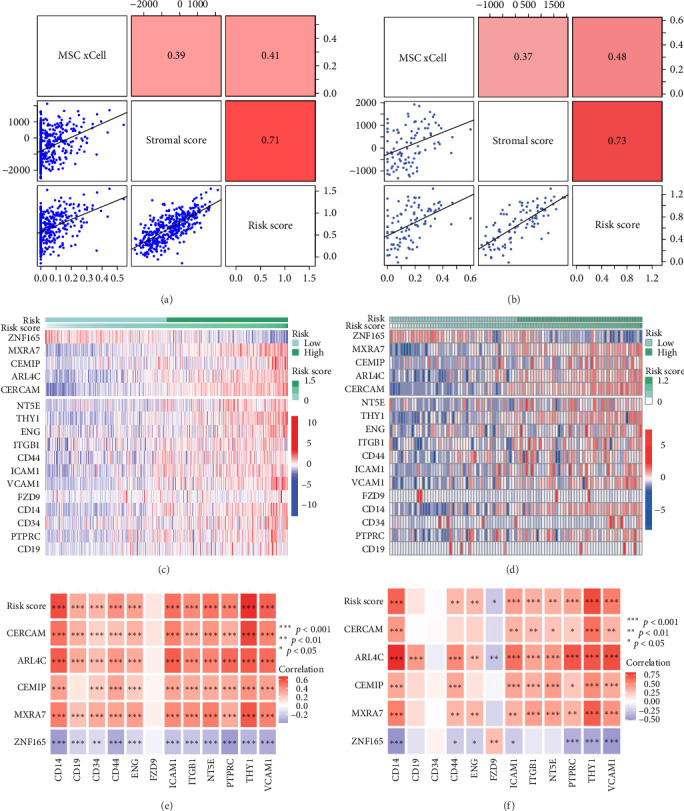
(A, B) Illustrations of Spearman's correlation assessments demonstrating a significant positive correlation between the MSC risk score and both stromal scores and MSC infiltrations in the TCGA-BLCA (A) and GSE31684 (B) cohorts. (C, D) Expression heat maps revealing distinct stratification patterns for five pivotal MSC genes in relation to the MSC risk score among TCGA-BLCA (C) and GSE31684 (D) subjects. (E, F) Documentation indicating a tangible positive correlation between the MSC risk score and the five signature genes, supported by literature addressing MSC markers in the TCGA-BLCA (E) and GSE31684 (F) groups. MSC, mesenchymal stem cell; TCGA-BLCA, The Cancer Genome Atlas Urothelial Bladder Carcinoma.

To validate the robustness of the MSC model as a predictor of MSC infiltrations, we conducted Spearman's correlation analyses between the MSC risk score and both the stromal score and MSC abundances predicted by xCell. The results consistently demonstrated a strong correlation between the MSC risk score, MSC infiltrations, and the stromal score across the TCGA-BLCA (Figure 5A) and GSE31684 (Figure 5B) cohorts. We examined the relationship between the MSC risk score, the expression levels of the five pivotal genes, and various MSC markers, revealing a strong positive correlation consistent across both cohorts, TCGA-BLCA (Figure 5C,E), and GSE31684 (Figure 5D,F).

### 3.6. Comparison of Chemotherapy and Immunotherapy Sensitivity among Different MSC-Risk Groups

**Figure 6:**
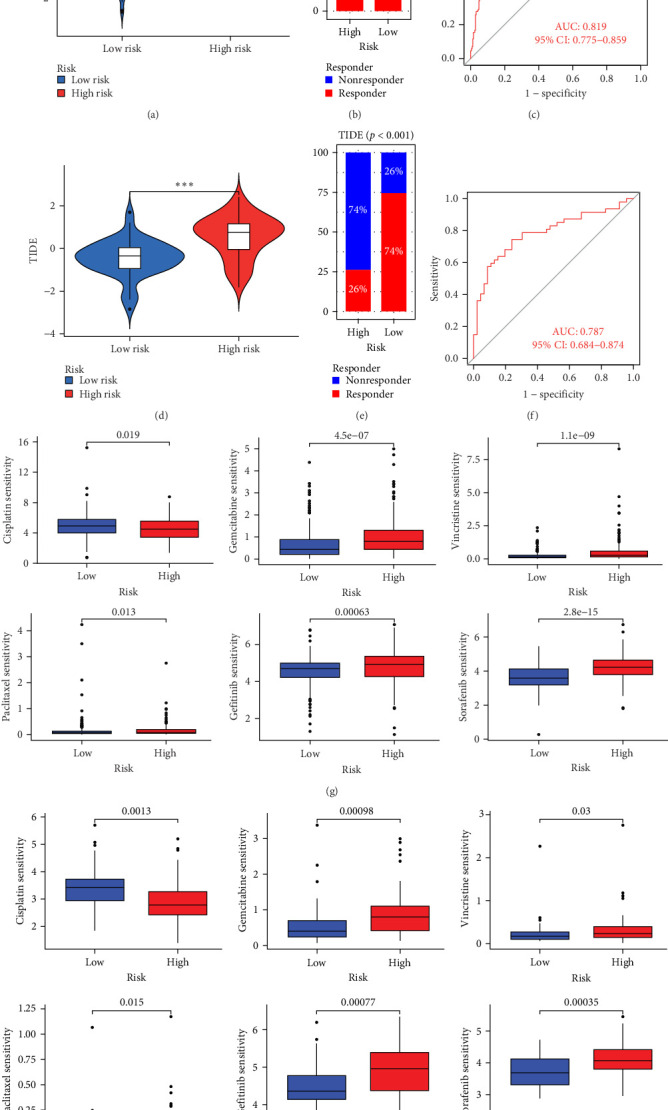
(A, D) Elevated TIDE scores observed in high-risk groups compared to low-risk counterparts in both the TCGA-BLCA (A) and GSE31684 datasets (D). (B, E) A notable skew toward immunotherapeutic responsiveness is evident in the low-MSC-risk category relative to the high-MSC-risk group, as illustrated in the TCGA-BLCA (B) and GSE31684 (E) datasets. (C, F) Area under the curve (AUC) values of 0.819 and 0.787 are reported for TCGA-BLCA (C) and GSE31684 (F), respectively. (G, H) Wilcoxon analyses reveal a significant inclination toward heightened sensitivity to therapeutic agents, including gemcitabine, vincristine, paclitaxel, gefitinib, and sorafenib, in bladder cancer patients with high-risk profiles and elevated MSC scores. Conversely, an increased sensitivity to cisplatin characterizes the low-MSC score subsets, a trend consistently observed across both TCGA-BLCA (G) and GSE31684 (H) patient cohorts. *⁣*^*∗∗∗*^*p* < 0.001. CI, confidence interval; MSC, mesenchymal stem cell; TCGA-BLCA, The Cancer Genome Atlas Urothelial Bladder Carcinoma; TIDE, tumor immune dysfunction and exclusion.

The applicability of the MSC risk score as a predictor of immunotherapy efficacy in BC patients was evaluated using the TIDE method, which showed a significantly higher TIDE score in the high-risk subgroup compared to the low-risk cohort in both the TCGA-BLCA and GSE31684 datasets (*p*  < 0.001; Figure 6A,D). A detailed analysis revealed that patients in the low-MSC-risk group exhibited a significantly higher responsiveness to immunotherapy compared to those in the high-MSC-risk group (*p*  < 0.001; Figure 6B,E). The efficacy assessment was further supported by AUC values, showing 0.819 (95% CI: 0.775–0.859) for the TCGA-BLCA dataset (Figure 6C) and 0.787 (95% CI: 0.684–0.874) for the GSE31684 dataset (Figure 6F), highlighting the robust performance of our MSC model in predicting immunotherapy response. The IC50 values of various drugs were estimated using data from the GDSC database. Wilcoxon analyses revealed that BC patients classified in the high-risk group, with elevated MSC scores, exhibited heightened sensitivity to gemcitabine, vincristine, paclitaxel, gefitinib, and sorafenib. Conversely, the low-MSC score subgroup demonstrated increased sensitivity to cisplatin, a trend consistently observed in both the TCGA-BLCA (Figure 6G) and GSE31684 (Figure 6H) cohorts. These findings highlight the predictive capability of the MSC model regarding drug responsiveness in BC.

### 3.7. GSEA Performed on the Five-Gene MSC Signature

**Figure 7:**
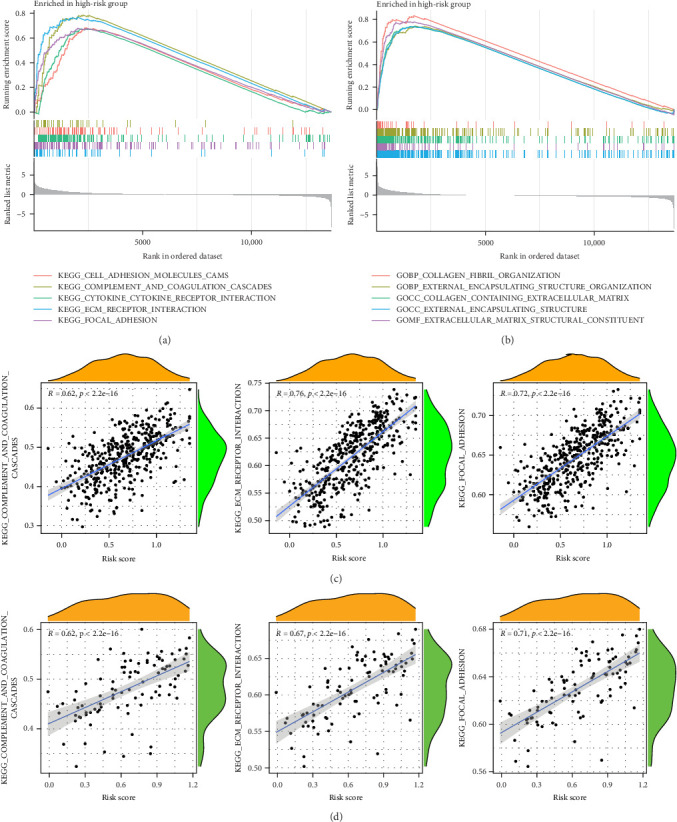
(A, B) Distinct stratifications in gene set enrichment analysis (GSEA) highlight differences in KEGG pathways (A) and hallmark gene sets (B) when comparing high- versus low-MSC risk groups. (C, D) Results from single-sample GSEA (ssGSEA) demonstrate a direct positive correlation between the MSC risk score and enrichment levels related to ECM–receptor interactions, focal adhesion, and the complexities of cytokine–receptor interactions, a trend consistently observed in both the TCGA-BLCA (C) and GSE31684 (D) cohorts. ECM, extracellular matrix; KEGG, Kyoto Encyclopedia of Genes and Genomes; MSC, mesenchymal stem cell; TCGA-BLCA, The Cancer Genome Atlas Urothelial Bladder Carcinoma.

To enhance understanding of the functional implications linked to the MSC signature, GSEA was performed on the TCGA-BLCA and GSE31684 datasets, comparing high- and low-MSC-risk groups. The results revealed significant enrichment in key KEGG signaling pathways, including ECM–receptor interaction, focal adhesion, and cytokine–cytokine receptor interactions (Figure 7A). Additionally, genes within the high-MSC-risk group showed notable enrichment in hallmark gene sets associated with external encapsulating structure organization and collagen fibril organization (Figure 7B). The ssGSEA results demonstrated a significant positive correlation between the MSC risk score and the enrichment scores of ECM–receptor interaction, focal adhesion, and cytokine–cytokine receptor interactions in both TCGA-BLCA (Figure 7C) and GSE31684 (Figure 7D), highlighting the intricate functional dynamics associated with elevated MSC risk classifications.

### 3.8. Validation of Key Genes Using the HPA Databases

**Figure 8:**
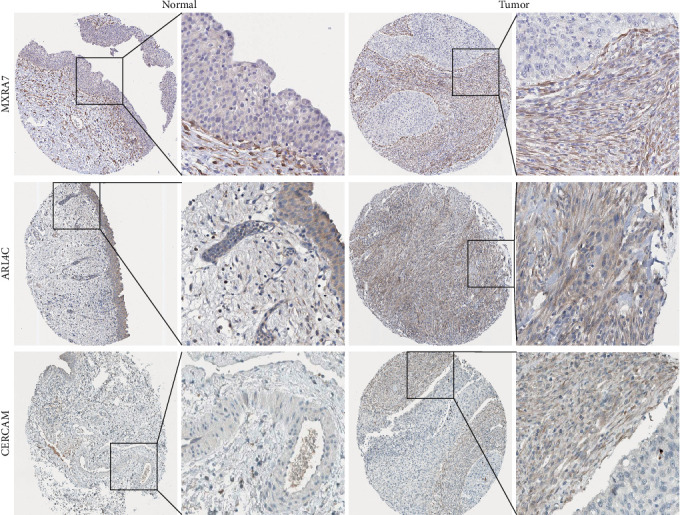
Protein expressions of MCRA7, ARL4C, and CERCAM in bladder cancer specimens from the Human Protein Atlas database.

To investigate the protein expression patterns of the MSC signature genes, we analyzed IHC images from the HPA database. The results revealed strong staining of matrix remodeling-associated 7 (MXRA7), ADP-ribosylation factor-like 4C (ARL4C), and cerebral endothelial cell adhesion molecule (CERCAM) in the BC stroma (Figure 8), indicating their potential as specific markers for MSCs.

### 3.9. Possible Targets and Molecular Docking of MSC-Related Genes in BC

**Figure 9:**
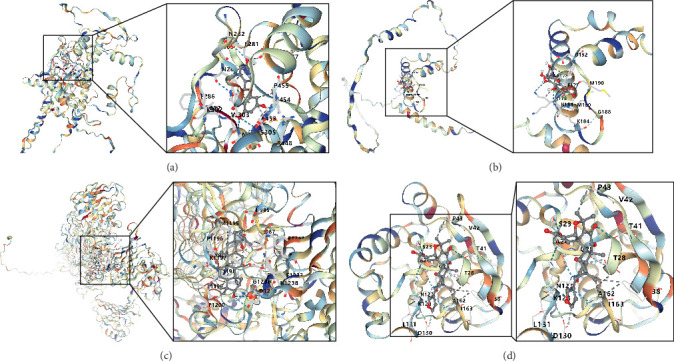
Molecular docking: (A) ZNF165-kaempferol, (B) MXRA7-quercetin, (C) CEMIP-mairin, and (D) ARL4C-limonin diosphenol.

In this study, we conducted further investigation into the drugs that effectively targeted five MSC-related genes in patients with BC. Our findings revealed that the drugs corresponding to four genes exhibited the potential to specifically impact BC (ZNF165, Haijinsha, Kuxingren, and Kunbu et al.; MXRA7, Chuanlianzi, Dazao, and Gehua et al.; CEMIP, Diercao, Maoyancao, and Muxiang et al.; and ARL4C, Baixianpi, Baihe, and Beiliujinu et al.), as evidenced by the data presented in Table S1. Subsequently, we selected the most promising drugs and their corresponding genes (ZNF165, Haijinsha; MXRA7, Chuanlianzi; CEMIP, Diercao; and ARL4C, Baixianpi) for detailed analysis. The TCMSP database was utilized to analyze the primary chemical constituents of the target drugs Haijinsha, Chuanlianzi, Diercao, and Baixianpi (Table 1). Subsequently, the drug exhibiting the highest OB (%) value was selected for molecular docking, aiming to validate the potential interaction between the drug and the target. The results of molecular docking demonstrated that kaempferol exhibited favorable docking with ZNF165 (Vina score = −7.7) (Figure 9A), quercetin exhibited favorable docking with MXRA7 (Vina score = −6.7) (Figure 9B), mairin exhibited favorable docking with CEMIP (Vina score = −7.6) (Figure 9C), and limonin diosphenol exhibited favorable docking with ARL4C (Vina score = −8.3) (Figure 9D). The stability observed in molecular docking experiments suggests that these compounds hold promise as effective therapeutic agents for targeting MSCs in the TME.

## 4. Discussion

To our knowledge, this study is the first to establish an MSC-related signature to predict prognosis and therapeutic responses in BC. We utilized WGCNA to estimate MSC and stromal infiltrations in BC. We identified hub modules that exhibited the strongest correlation with stromal and MSC infiltrations. Subsequently, through the application of univariate and LASSO Cox regression analyses, we identified ZNF165, MXRA7, CEMIP, ARL4C, and CERCAM as prognostic MSC markers. Furthermore, we constructed a five-gene MSC signature that demonstrated the ability to predict prognosis and therapeutic responses in BC. Our findings suggest that the MSC model may represent a novel therapeutic approach targeting MSCs in BC.

The TME is intricately composed, with a myriad of CCs beyond immune cells, encompassing a variety of stromal cells like MSCs, fibroblasts, endothelial cells, and pericytes, each contributing to the complex milieu [34]. Despite the existence of prognostic models associated with cancer-associated fibroblasts [35], a dedicated model centered on MSCs is currently lacking. The propensity of MSCs to be recruited to tumor sites has been a consistent observation across diverse tumor types [36–44]. This phenomenon of MSC recruitment is not just incidental but plays a pivotal role in determining the trajectory of tumor progression. Existing literature accentuates the dual role MSCs can potentially play; however, a substantial body of evidence inclines toward their protumorigenic influence, where they are implicated in augmenting tumor growth, promoting angiogenesis, facilitating metastasis, and instigating resistance to therapeutic drugs [26]. In alignment with this, the findings from the current research elucidate a direct correlation between elevated MSC infiltration and diminished OS, underscoring the imperative to understand this dynamic for enhanced therapeutic strategies.

MSCs exert a pivotal influence on the chemosensitivity of tumor entities, driven by their multifaceted secretory profile. Insight from a murine BC model revealed that bone marrow–derived MSC exosomes carry miR-23b [45]. This discrete microRNA is instrumental in counteracting the myristoylated alanine-rich C-kinase substrate, an essential protein kinase C substrate. This strategic interference catalyzes a dormancy stage in metastatic niche-residing cancer stem cells, fostering a robust defense mechanism against the chemotherapy agent docetaxel [45]. In a related observation, the therapeutic potential of platinum compounds witnessed a stark nullification with the intravenous introduction of bone marrow MSCs (BM-MSCs), as documented across three experimental subcutaneous tumor paradigms [46]. Upon encountering platinum-based chemotherapy, BM-MSCs respond by discharging polyunsaturated fatty acids. These specific secretions are recognized for their role in shielding various cancer forms, namely, colon, lung, and breast carcinomas, against the cytotoxic influence of platinum-derived drugs [46]. The current investigation accentuates the relevance of a five-gene MSC signature in forecasting the responsiveness to an array of chemotherapeutic agents, encompassing cisplatin, gemcitabine, vincristine, and paclitaxel. This genetic constellation holds substantial promise in refining therapeutic strategies, anchored by its predictive precision.

The landscape of BC treatment has undergone substantial transformation with the advent of immune checkpoint inhibitors (ICIs). These agents have proven effective, particularly for a subset of patients, instilling a durable immunologic memory [47]. However, the response rate of UC to ICIs hovers around 15%–21% in the context of unselected metastatic cases [48]. Distinct from targeted therapies, the realm of ICI treatment still grapples with the absence of predictive molecular biomarkers, rendering the identification of likely responders a significant clinical challenge. Given MSCs' established role in modulating both innate and adaptive immune elements, conditioning the TME for facilitating neoplastic proliferation and progression [49], the impetus has shifted toward leveraging MSC-based insights for biomarker discovery. This strategy holds promise in refining patient screening processes for ICI therapy, contributing to more personalized therapeutic protocols. Reinforcing this perspective, findings from the present analysis indicate that an MSC-related prognostic construct holds efficacy in anticipating responses to ICI, marking a potential paradigm shift in oncological personalization.

The therapeutic landscape in cancer research has recently pivoted toward the TME, recognizing its crucial influence on tumor behavior and treatment outcomes [17]. This investigation pinpointed ZNF165, MXRA7, CEMIP, ARL4C, and CERCAM as promising prognostic indicators within the context of MSCs in BC, underscoring their potential as innovative therapeutic intervention points. ZNF165 emerges as a constituent of the Kruppel-like cadre of zinc-finger transcription modulators, notorious for its upregulation across a spectrum of malignancies encompassing urinary bladder transitional cell carcinoma [50], gastrointestinal cancer [51], and hepatocellular carcinoma [52], triple-negative breast cancer [53]. Interaction paradigms between ZNF165 and SMAD3 underscore its regulatory capacity on transforming growth factor beta (TGF-β)-responsive gene transcription [54], with distinct pathways elucidated in the proliferation and motility augmentation within hepatocellular carcinoma contexts, notably via tryptophan/kynurenine/AhR/CYP1A1 axis engagement [52]. CEMIP, documented across various tumor types, correlates with enhanced cellular motility, invasiveness, and fortified resistance against pharmacological interventions [55–57]. Specific interactions with Bip within the endoplasmic domain, precipitating Ca^2+^ liberation and subsequent PKCα signal transduction, have been implicated in breast cancer metastasis [55]. A distinct scaffolding function was observed in CEMIP, mediating GRAF1 and MIB1 coordination, thereby instigating metastatic events in colorectal cancer through CDC42/MAPK pathway activation [58]. Similarly, ARL4C, abundant within neoplastic tissues, perpetrates its tumorigenic influence across various cancer forms including, but not limited to, colorectal, pulmonary, and hepatic carcinomas [59–61]. Mechanistic insights link its activity to Rac1 stimulation, Rho attenuation, and PIK3CD amplification [59, 61], with further implications in tumor–stroma interplay impacting pancreatic cancer resilience and expansion [62]. In a parallel vein, CERCAM's dysregulated expression profiles in diverse cancers hint at its contributory role in oncogenesis and malignant transformation [63, 64]. Empirical data accentuate CERCAM's role in fostering cellular survival, DNA synthetic processes, and invasive potential specifically within BC matrices [65]. In summation, the molecules ZNF165, MXRA7, CEMIP, ARL4C, and CERCAM collectively represent prospective therapeutic nexus points in impeding tumor advancement. However, a comprehensive understanding of the exact mechanistic pathways endorsing BC proliferation via these molecules necessitates further exploration.

Acknowledging the limitations of this study is essential. Our research primarily utilized a retrospective bioinformatics approach, drawing from two publicly available gene expression datasets. This highlights the need for robust validation of the prognostic and therapeutic significance of the MSC model through multicenter and prospective studies. Additionally, the specific biological roles of the MSC signature biomarkers in BC should be confirmed through detailed molecular and animal experiments. Despite these limitations, our findings provide a valuable foundation for future investigations into the role of MSCs in BC.

## 5. Conclusions

The current five-gene prognostic MSC signature demonstrates reliability in predicting outcomes and effectively assessing responses to clinical chemotherapy and immunotherapy in BC patients. The genes ZNF165, MXRA7, CEMIP, ARL4C, and CERCAM show promise as therapeutic targets for anti-MSC interventions.

## Figures and Tables

**Table 1:** Composition and relevant information of drugs corresponding to hub genes.

Gene	Medicine	Molecule ID	Molecule name	OB (%)	DL	HL
ZNF165	Haijinsha	MOL001506	Kaempferol	41.88	0.24	14.74
	—	MOL001689	Acacetin	34.97	0.24	17.25
	—	MOL001790	Linarin	39.84	0.71	16.07
	—	MOL002879	Diop	43.59	0.39	3.6
	—	MOL002881	Diosmetin	31.14	0.27	16.34
	—	MOL002882	[(2R)-2,3-Dihydroxypropyl] (Z)-octadec-9-enoate	34.13	0.3	5.19
	—	MOL002883	Ethyl oleate (NF)	32.4	0.19	4.85
	—	MOL000296	Hederagenin	36.91	0.75	5.35
	—	MOL000358	Beta-Sitosterol	36.91	0.75	5.36
	—	MOL000422	Supraene	33.55	0.42	2.72

MXRA7	Chuanlianzi	MOL001494	Quercetin	46.43	0.28	14.4
	—	MOL001495	Ethyl linolenate	46.1	0.2	6.2
	—	MOL002045	Stigmasterol	43.41	0.76	6.29
	—	MOL002047	Melianone	40.73	0.81	6.92
	—	MOL002048	Nimbolidin D	30.38	0.53	10.82
	—	MOL002053	Nimbolin A	32.11	0.34	7.33
	—	MOL002056	(E)-3-[(2S,3R)-2-(4-hydroxy-3-methoxy-phenyl)-7-methoxy-3-methylol-2,3-dihydrobenzofuran-5-yl]acrolein	54.74	0.4	8.03
	—	MOL002058	40957-99-1	57.2	0.62	2.04
	—	MOL000098	Mandenol	42	0.19	5.39

CEMIP	Diercao	MOL000211	Mairin	55.38	0.78	8.87
	—	MOL000358	Beta-Sitosterol	36.91	0.75	5.36
	—	MOL000359	Sitosterol	36.91	0.75	5.37
	—	MOL000422	Kaempferol	41.88	0.24	14.74
	—	MOL006772	Poriferasterol monoglucoside_qt	43.83	0.76	5.32
	—	MOL007879	Tetramethoxyluteolin	43.68	0.37	15.45
	—	MOL007880	3,5,7,3,5-Pentahydroxy flavonol	63.64	0.24	1.84
	—	MOL000098	Quercetin	46.43	0.28	14.4

ARL4C	Baixianpi	MOL006236	Limonin diosphenol	91.69	0.55	−3.24
	—	MOL006257	Dictamnusine_qt	69.27	0.19	−11.1
	—	MOL006235	Dasycarpamin	59.14	0.21	2.19
	—	MOL006272	9alpha-Hydroxyfraxinellone-9-o-beta-d-glucoside	55.52	0.62	−6.45
	—	MOL006244	(1R,4R,6S,8aR)-1-(3-Furyl)-4,6-dihydroxy-5,8a-dimethyl-4,6,7,8-tetrahydro-1H-isochromen-3-one	49.09	0.19	−11.68
	—	MOL006233	3′-O-methyl taxifolin	48.36	0.3	14.86
	—	MOL000098	Quercetin	46.43	0.28	14.4
	—	MOL006270	Tirucallane	44.02	0.75	5.97
	—	MOL013352	Obacunone	43.29	0.77	13.04
	—	MOL006268	Preskimmianine	42.14	0.21	1.64
	—	MOL002663	Skimmianin	40.14	0.2	−2.43
	—	MOL005043	Campest-5-en-3beta-ol	37.58	0.71	4.43
	—	MOL000358	Beta-Sitosterol	36.91	0.75	5.36
	—	MOL000006	Luteolin	36.16	0.25	15.94
	—	MOL006262	7alpha-Acetylobacunol	36.08	0.71	−11.88
	—	MOL006271	Trichirubine,b	35.51	0.27	19.24
	—	MOL006261	Isomaculosidine	31.99	0.2	12.96
	—	MOL000173	Wogonin	30.68	0.23	17.75

Abbreviations: DL, drug likeness; HL, drug half-life; OB, oral bioavailability.

## Data Availability

Publicly available datasets were analyzed in this study. This data can be found here: TCGA (https://portal.gdc.cancer.gov/repository) and GEO (https://www.ncbi.nlm.nih.gov/geo/) database. The data generated and analyzed in this study are available from the corresponding author upon reasonable request.

## Supporting Information

Supporting Information Table S1. Information on target and natural compounds.
