# Supplementary material for: Identification of a Novel Mesenchymal Stem Cell–Related Signature for Predicting the Prognosis and Therapeutic Responses of Bladder Cancer
Source: Stem Cells Int. 2024 Nov 15;2024:6064671. doi: 10.1155/sci/6064671 (PMC11611448; doi:10.1155/sci/6064671)
Supplement: Supporting Information — Table S1. Information on target and natural compounds. [file 6064671.f1.docx]

Supplement table 1. Information on target and natural compounds

| **Gene** | **Herb id** | **Pinyin name** | **Latin name** | **English name** | **Class in English** | **P value** | **FDR(BH)** | **Relationship** |
| --- | --- | --- | --- | --- | --- | --- | --- | --- |
| ZNF165 | SMHB00088 | Dasuan | Allii Sativi Bulbus | Garlic | Medicinal For Detoxification、Parasiticide、Drying Dampness And Relieving Itching | 0.034953 | 0.076563 | By_ingredient |
|  | SMHB00157 | Haijinsha | Lygodii Spora | spore of Japanese climbing Fern | Diuretic Dampness Excreting Drugs | 0.025974 | 0.067674 | By_ingredient |
|  | SMHB00230 | Kuxingren | Armeniacae Semen Amarum | bitter Apricot seed | Antitussive Antiasthmetics | 0.038303 | 0.079882 | By_ingredient |
|  | SMHB00232 | Kunbu | Laminariae Thallus Eckloniae Thallus | Kelp or Tangle | Phlegresolving Medicine | 0.023041 | 0.064993 | By_ingredient |
|  | SMHB00283 | Mohanlian | Ecliptae Herba | all-grass of Yerbadetajo | Yin-Tonifying Medicinal | 0.0201 | 0.061297 | By_ingredient |
|  | SMHB00360 | Shanzha | Crataegi Folium | Crataegi Folium | Blood Activating Stasis Removing Drugs | 0.032715 | 0.074676 | By_ingredient |
|  | SMHB00658 | Lurong | Cervi Cornu Pantotrichum | None | Tonifying And Replenishing Medicinal | 0.005516 | 0.037845 | By_ingredient |
|  | SMHB00681 | Fengfang | Vespae Nidus | None | Toxin-Attacking And Worm-Expelling And Itch-Relieving Medicinal | 0.00712 | 0.041619 | By_ingredient |
|  | SMHB00702 | Shexiang | Moschus | None | Resuscitative Stimulant; Resuscitative Medicinal | 0.011919 | 0.050166 | By_ingredient |
| MXRA7 | SMHB00072 | Chuanlianzi | Toosendan Fructus | Szechwan Chiberry Fruit | Qi Regulating Drugs | 0.005067 | 0.036294 | By_ingredient |
|  | SMHB00090 | Dazao | Jujubae Fructus | Jujube Chinese date | Qi Reinforcing Drugs | 0.009674 | 0.046911 | By_ingredient |
|  | SMHB00139 | Gehua | Flos Puerariae Lobatae | flower bud of lobed kudzuvine | Pungent Cool Diaphoretics | 0.008369 | 0.044289 | By_ingredient |
|  | SMHB00319 | Qianhu | Peucedani Radix | root of Grand Hogfennel | Phlegresolving Medicine | 0.006948 | 0.041339 | By_ingredient |
|  | SMHB00386 | Suanzaoren | Ziziphi Spinosae Semen | Spine Date Seed | Tranguilizing Medicinal | 0.004376 | 0.034202 | By_ingredient |
|  | SMHB00412 | Weilingcai | Potentiliae Chinensis Herba | Chinese Cinquefoil | Antipyretic Detoxicate Drugs | 0.003148 | 0.030031 | By_ingredient |
|  | SMHB00499 | Gegen | Puerariae Lobatae Radix | root of lobed kudzuvine | Pungent-Warm Exterior-Releasing Medicinal | 0.003954 | 0.032859 | By_ingredient |
| CEMIP | SMHB00088 | Dasuan | Allii Sativi Bulbus | Garlic | Medicinal For Detoxification、Parasiticide、Drying Dampness And Relieving Itching | 0.046333 | 0.087694 | By_ingredient |
|  | SMHB00100 | Diercao | Herba Hyperici Japonici | all - grass of Japanese St. Johnswort | Diuretic Dampness Excreting Drugs | 0.001754 | 0.022172 | By_ingredient |
|  | SMHB00275 | Maoyancao | None | Lunulatae Herba | Phlegresolving Medicine | 0.000215 | 0.005611 | By_ingredient |
|  | SMHB00292 | Muxiang | Aucklandiae Radix | Common Aucklandia Root | Qi Regulating Drugs | 0.001975 | 0.023314 | By_ingredient |
|  | SMHB00313 | Jicai | Herba Capsellae | all-grass of Shepherdspurse | Diuretic Dampness Excreting Drugs | 1.05E-05 | 0.000602 | By_ingredient |
|  | SMHB00353 | Shaji | Hippophae Fructus | fruit of seabuckthorn | Phlegresolving Medicine | 0.004285 | 0.034202 | By_ingredient |
|  | SMHB00619 | Fulingpi | Poriae Cutis | None | Diuretic Dampness Excreting Drugs | 0.037754 | 0.07941 | By_ingredient |
|  | SMHB00672 | Suoyang | Cynomorii Herba | None | Tonifying And Replenishing Medicinal | 0.007653 | 0.043151 | By_ingredient |
| ARL4C | SMHB00025 | Baixianpi | Dictamni Cortex | Dictamni Cortex | Heat-Clearing And Dampnessdrying Medicinal | 0.04768 | 0.088952 | By_ingredient |
|  | SMHB00029 | Baihe | Lilii Bulbus | Bulb of Greenish Lily | Yin-Tonifying Medicinal | 0.038042 | 0.079722 | By_ingredient |
|  | SMHB00039 | Beiliujinu | Siphonostegiae Herba | Siphonostegiae Herba | Blood Activating Stasis Removing Drugs | 0.020882 | 0.062581 | By_ingredient |
|  | SMHB00052 | Cangzhu | Atractylodis Rhizoma | Rhizome of Swordlike Atractylodes | Dampness Removing Drugs | 0.048672 | 0.089539 | By_ingredient |
|  | SMHB00079 | Chuanxinlian | Andrographis Herba | all-grass of Common Androgrphis | Antipyretic Detoxicate Drugs | 0.00128 | 0.018145 | By_ingredient |
|  | SMHB00082 | Congbai | Bulbus Allii Fistulosi | Allium bulb, Wild scallion, Chinese green onion | Pungent-Warm Exterior-Releasing Medicinal | 0.04204 | 0.083573 | By_ingredient |
|  | SMHB00087 | Daqingye | Isatidis Folium | Isatis leaf | Antipyretic Detoxicate Drugs | 0.036706 | 0.078279 | By_ingredient |
|  | SMHB00099 | Dengxincao | Junci Medulla | pith of Common Rush | Diuretic Dampness Excreting Drugs | 0.034363 | 0.075917 | By_ingredient |
|  | SMHB00120 | Fanxieye | Sennae Folium | Sen leaf | Offensive Purgative Medicinal | 0.038042 | 0.079722 | By_ingredient |
|  | SMHB00142 | Gouguye | Ilicis Cornutae Folium | Folium Ilicis Cornutae | Asthenic Heat Dispelling Drugs | 0.048341 | 0.089406 | By_ingredient |
|  | SMHB00154 | Guizhencao | Herba Bidentis | all-grass of Spanishneedles | Antipyretic Detoxicate Drugs | 0.026632 | 0.068628 | By_ingredient |
|  | SMHB00157 | Haijinsha | Lygodii Spora | spore of Japanese climbing Fern | Diuretic Dampness Excreting Drugs | 0.038709 | 0.080112 | By_ingredient |
|  | SMHB00181 | Huajuhong | Citri Grandis Exocarpium | Pummelo Peel | Qi Regulating Drugs | 0.037374 | 0.07904 | By_ingredient |
|  | SMHB00188 | Huangqin | Scutellariae Radix | root of Baikal skullcap | Heat-Clearing And Dampnessdrying Medicinal | 0.004054 | 0.033163 | By_ingredient |
|  | SMHB00193 | Jishiteng | None | Paederiae Scandentis Herba | Digestants | 0.04503 | 0.086629 | By_ingredient |
|  | SMHB00206 | Jinqiaomai | Fagopyri Dibotryis Rhizoma | Wild Buckwheat Rhizome | Antipyretic Detoxicate Drugs | 0.04337 | 0.084964 | By_ingredient |
|  | SMHB00226 | Kudiding | Corydalis Bungeanae Herba | all - grass of Bunge Corydalis | Antipyretic Detoxicate Drugs | 0.025957 | 0.067674 | By_ingredient |
|  | SMHB00241 | Lianqiao | Forsythiae Fructus | Weeping Forsythia Capsule | Antipyretic Detoxicate Drugs | 0.003184 | 0.030288 | By_ingredient |
|  | SMHB00253 | Longliye | Sauropi Folium | Aauropi Folium | External Medicinal (Draw Out Toxin, Resolve Putridity) | 0.034363 | 0.075917 | By_ingredient |
|  | SMHB00255 | Lugen | Phragmitis Rhizoma | Reed rhizome | Fire Purging Drugs | 0.037708 | 0.079376 | By_ingredient |
|  | SMHB00259 | Luobumaye | Apocyni Veneti Folium | Apocyniveneti Folium | Liver-Pacifying Wind-Extinguishing Medicinal | 0.027643 | 0.069764 | By_ingredient |
|  | SMHB00275 | Maoyancao | None | Lunulatae Herba | Phlegresolving Medicine | 0.024944 | 0.066918 | By_ingredient |
|  | SMHB00280 | Mihoutaogen | None | Actinidia Chinensis Planch | Antipyretic Detoxicate Drugs | 0.022237 | 0.064234 | By_ingredient |
|  | SMHB00289 | Muhudie | Oroxyli Semen | Indian Trumpetflower Seed | Antipyretic Detoxicate Drugs | 0.04204 | 0.083573 | By_ingredient |
|  | SMHB00308 | Pangdahai | Sterculiae Lychnophorae Semen | Boat-fruited Sterculia Seed | Phlegresolving Medicine | 0.026969 | 0.069043 | By_ingredient |
|  | SMHB00309 | Peilan | Eupatorii Herba | Fortune Eupatorium Herb | Dampness Removing Drugs | 0.048341 | 0.089406 | By_ingredient |
|  | SMHB00334 | Qumai | Dianthi Herba | all-grass of Lilac Pink | Diuretic Dampness Excreting Drugs | 0.029327 | 0.071189 | By_ingredient |
|  | SMHB00339 | Roudoukou | Myristicae Semen | Nutmeg | Astringent Medicinal | 0.044366 | 0.085918 | By_ingredient |
|  | SMHB00378 | Shuqucao | None | Gphalii Affinis Herba | Phlegresolving Medicine | 0.03971 | 0.08125 | By_ingredient |
|  | SMHB00379 | Shuifeiji | Silybi Fructus | Silybum Marianum | Antipyretic Detoxicate Drugs | 0.032352 | 0.074261 | By_ingredient |
